# Results of interferon-free treatment for HCV-infected patients with decompensated cirrhosis from a Brazilian real-life cohort

**DOI:** 10.1097/MD.0000000000030097

**Published:** 2022-09-02

**Authors:** Lívia Victor, Renata Perez, Flávia Fernandes, Juliana Piedade, Cristiane A. Villela-Nogueira, Gustavo Pereira

**Affiliations:** a School of Medicine, Internal Medicine Department, Hepatology Division, Federal University of Rio de Janeiro, Rio de Janeiro, Brazil; b Gastroenterology and Hepatology Unit, Bonsucesso Federal Hospital, Rio de Janeiro, Brazil; c School of Medicine, Estácio de Sá University, Rio de Janeiro, Brazil.

**Keywords:** decompensated cirrhosis, efficacy, hepatitis C virus, interferon-free treatment, real-life study, sustained virological response

## Abstract

Real-life data on the HCV treatment with direct-acting agents in patients with decompensated cirrhosis are scarce. Study to investigate the effectiveness and safety of sofosbuvir-containing regimens in a prospective cohort of patients with HCV decompensated cirrhosis. A total of 150 patients were enrolled (64% male, 84% genotype 1 with a mean age of 61 ± 9 years). The median MELD was 12, and 79% were Child-PughB. Most patients were treated with sofosbuvir and daclatasvir (98%) with ribavirin in 27%. The overall intention to treat SVR12 was 91% (137/150). The most frequent adverse event was anemia (17%), 73% associated with ribavirin. Twenty-one (14%) patients experienced renal dysfunction, 81% AKI I, and 1 discontinued treatment. Thirty-five (23%) patients presented at least 1 infectious episode, mainly respiratory tract infection (29%). Thirty-three patients (22%) had at least 1 episode of cirrhosis decompensation throughout treatment, particularly worsening of previous ascites in 19%. Nine patients died, and among those, 7 patients died from sepsis. The probability of decompensation in 28, 90 and 180 days was 4%, 19% and 25%. During treatment, infection (OR 2.24; 95 CI 1.09–4.61; *P* = .03) was a predictor of cirrhosis decompensation, and baseline MELD and CHILD ≥ B8 were both associated with infection. In decompensated cirrhosis, the overall virological response was high with mild adverse events. However, this population had a high frequency of liver-associated decompensation and infections.

## 1. Introduction

Hepatitis C virus (HCV) infection is a global health concern with an estimated worldwide disease prevalence of 1%.^[1]^ In Brazil, the estimated prevalence of antiHCV-positive individuals is 0.53%, with a total of 632,000 with detected HCV-RNA.^[2]^ In the absence of treatment, chronic hepatitis C is an insidious disease with an average rate of 20% progression to cirrhosis over time.^[1]^ After an initial episode of hepatic decompensation, the risk of death in the following 12 months is 15 to 20%.^[1]^

Until recently, Interferon-based therapy for hepatitis C (HCV) has been contraindicated to patients with advanced cirrhosis primarily due to safety concerns and efficacy.

The availability of direct-acting antivirals (DAA) effective against HCV chronic infection has radically changed the scenario of decompensated cirrhotic patients.^[3–5]^ These treatments have shown to be safe and effective in clinical trials when used in this population.^[6–8]^ Some further real-life also confirmed these encouraging results.^[9–19]^

Nevertheless, most studies on this topic were randomized controlled trials, with scarce data on real-life cohorts.^[10,12,13,19]^ Also, this population is prone to the development of complications of cirrhosis, which was explicitly a significant issue in patients treated with interferon-based^[20]^ and simeprevir-containing regimens.^[21,22]^ Complications of cirrhosis may impair the ability to maintain antiviral treatment (due to limited level of consciousness in patients with hepatic encephalopathy or limitations to drug administration in patients with kidney function impairment) and survival. Despite this, there is a paucity of data regarding the frequency and clinical relevance of complications of cirrhosis in decompensated patients treated with DAAs in real-life cohorts, mostly related to hepatocellular carcinoma and liver transplantation.^[11,19]^

Since data from Brazilian patients are scarce, our study aimed to investigate the effectiveness and safety of sofosbuvir-containing regimens for HCV in a real-life cohort of patients with decompensated cirrhosis.

## 2. Patients and Methods

### 2.1. Study design and patients

We conducted a prospective cohort study including decompensated cirrhotic outpatients treated in 2 Brazilian tertiary hospitals from October 2015 to November 2017. Patients were enrolled upon meeting the following inclusion criteria: adults (≥18 years old) with chronic HCV infection and decompensated cirrhosis, defined as Child-PughB/C or Child-Pugh A with a previous history of ascites, hepatic encephalopathy (HE) or variceal gastrointestinal hemorrhage. Exclusion criteria were: other associated liver disease such as HBV coinfection, autoimmune hepatitis, hepatosplenic schistosomiasis, HIV coinfection, previous liver transplantation and advanced HCC (Milan criteria C and D).

Patients were treated according to the Brazilian Ministry of Health guidelines as follows: genotype 1 and 4 patients received once-daily sofosbuvir (400 mg) and daclatasvir (60 mg) with or without ribavirin (weight-based dose, as tolerated) for 12 weeks if Child-Pugh A or 24 weeks if Child-Pugh B/C. Genotype 2 patients received once-daily sofosbuvir (400 mg) with ribavirin (weight-based dose, as tolerated) for 12 weeks. Genotype 3 patients received once-daily sofosbuvir (400 mg) and daclatasvir (60 mg) with or without ribavirin (weight-based dose, as tolerated) for 12 weeks.

Patients’ baseline characteristics were registered, including demographics data (age and gender), comorbidities (such as diabetes, essential arterial hypertension, chronic renal failure considering glomerular filtration rate <60 ml/min/1.73 m²), prior episodes of cirrhosis decompensation (including ascites, hepatic encephalopathy, upper gastrointestinal variceal hemorrhage and hepatocellular carcinoma) and hepatitis C genotype. Patients were scheduled for follow-up visits at the start of treatment, weeks 4, 8, 12 during treatment, end of treatment and 12 weeks after that when SVR was assessed. Clinical exam and laboratory tests performed at each visit included blood tests with renal and hepatic function: bilirubin, aspartate aminotransferase, alanine aminotransferase, albumin, hemoglobin, white blood count, platelets, INR, creatinine, sodium, presence of ascites, and hepatic encephalopathy. MELD and Child-Pugh scores were calculated.^[23,24]^

In addition, at each visit, we investigate, through clinical history and physical examination, episodes of arrhythmia, infections, and new or worsening episodes of hepatic decompensation such as variceal hemorrhage, ascites, and hepatic encephalopathy. If any changes were identified in the initial assessment, additional tests, such as electrocardiogram, laboratory tests and imaging tests, were necessary for diagnostic confirmation. The usual recommendation for hepatocellular carcinoma screening, such as performing ultrasonography every 6 months, was maintained, and the emergence of new nodules during the study was also recorded.

The IRB approved the study of both hospitals. All patients have signed the informed consent form.

### 2.2. Statistical analysis

Initially, demographic virologic and clinical characteristics at the beginning of treatment of the entire cohort were described. We used frequency and percentage for categorical variables and mean and standard deviation or median and range for the continuous ones. Comparisons between groups were performed using the chi-square test for categorical and Wilcoxon tests for continuous data.

The Child-Pugh score was analyzed as a categorical variable. We determined the best cut-off point of 8 to predict infection and decompensation using AUROC curves.

Two separate analyses were performed to identify bacterial infection or hepatic decompensation factors. The first development of any hepatic decompensation or bacterial infections mentioned before was considered the index event for statistical purposes.

Variables significantly associated with decompensation or infection were selected on univariate analysis. Multivariate analysis was done using stepwise backward logistic regression.

Due to the direct causal relationship between bacterial infections and liver decompensation, bacterial infections were included in the univariate analysis of factors associated with liver decompensation, but not the opposite. In all analyses, differences were considered significant at the level of 5%. Statistical analysis was performed using the IBM SPSS 21 for Windows program (SPSS^®^, IBM Inc, New York). All data will be available from the corresponding author upon demand.

## 3. Results

A total of 150 patients with HCV-related decompensated cirrhosis were enrolled. Nine patients died during the treatment, 1 patient underwent liver transplantation, and another was lost to follow-up. Outcome data at the end of treatment was available for 139 patients.

Baseline characteristics are shown in Table 1. Patients were mostly male (64%) with a mean age of 61 ± 9 years old. Over 63% were treatment-naive patients, 37% had been previously treated with pegylated interferon and ribavirin and of these, 18% were null responders, and 5 (9%) failed first-generation DAAs telaprevir and boceprevir when they had compensated cirrhosis. Diabetes and essential arterial hypertension were present in 39% and 48% of patients. Genotype 1 (84%) was the most common (G1a 42%; G1b 27%; G1 without subgenotype 15%). The median MELD score was 12 (7–24), and 79% were Child-Pugh B. Two patients had a history of hepatocellular carcinoma before treatment. At the beginning of treatment, 48% of patients presented or were treated for ascites, and 23% presented or received treatment for hepatic encephalopathy.

**Table 1:** Baseline characteristics of decompensated cirrhotic patients treated with Sofosbuvir-based regimens (n = 150).

Variables	Study population (n = 150)
Age (yr)	61 ± 9
Male gender	96 (64%)
Esophageal varices	123 (83%)
Comorbidities	
Diabetes	59 (39%)
Systemic arterial hypertension	72 (48%)
Chronic renal failure	10 (7%)
Kidney transplantation	3 (2%)
Previous cirrhosis decompensation	
Ascites	98 (65%)
Hepatic encephalopathy	44 (29%)
Variceal upper gastrointestinal hemorrhage	52 (35%)
Hepatocellular carcinoma	2 (1%)
Previous regular medications	
Propranolol	100 (67%)
Spironolactone	78 (52%)
Furosemide	42 (28%)
Lactulone	25 (17%)
Virological characteristics	
Genotype 1	126 (84%)
Genotype 2	3 (2%)
Genotype 3	18 (12%)
Genotype 4	3 (2%)
Clinical and laboratory characteristics at baseline	
Bilirubin (mg/dl)	1.7 ± 0.9
Aspartate aminotransferase (U/L)	98 ± 46
Alaline aminotransferase (U/L)	86 ± 51
Albumin (g/dl)	30 ± 0,5
Hemoglobin (g/dL)	12.6 ± 1.8
White blood cell count (×10^9^/mm³)	4.8 ± 1.9
Platelet count (×10^9^/mm³)	94 ± 57
RNI	1.3 ± 0.3
Creatinine (mg/dl)	1.0 ± 0.7
Sodium (mEq/L)	139 ± 4
MELD	12 ± 3
[scolor_start FADADD]≤[/scolor]10	36 (24%)
[scolor_start FADADD]≥[/scolor]14	42 (28%)
Child-Pugh	
A	20 (13%)
B	118 (79%)
C	12 (8%)

Most patients were treated with sofosbuvir and daclatasvir (98%). Ribavirin was prescribed in 73% of patients. Regarding genotypes, ribavirin was used by 71% (89/126) of genotype-1 patients, 100% (3/3) of genotype-2 patients, 83% (15/18) of genotype-3 patients, and 100% (3/3) of genotype patients 4. Two genotype-1 patients classified as Child-Pugh A were treated with sofosbuvir and simeprevir, and 1 genotype-2 patients were treated with sofosbuvir and ribavirin, according to the Brazilian guideline for the treatment of hepatitis C at the time of the study.

### 3.1. Virological response

Of 139 patients that completed treatment, only 2 had positive HCV-RNA at 12 weeks posttreatment and did not attain SVR. They were previous nonresponders to pegylated interferon and ribavirin. One was genotype 3, treated with 12 weeks of sofosbuvir, daclatasvir, and ribavirin. The other was a genotype 1b, treated with 24 weeks of the same regimen, with a negative HCV-RNA at the end of treatment, and relapsed 12 weeks later. No drug-drug interaction was observed in any of the patients who failed treatment. Therefore, the overall intention to treat SVR12 was 91% (137/150), and the per-protocol SVR rate was 98.6% (137/139).

### 3.2. Adverse events and death

The frequency of adverse events and liver-related decompensation are shown in Table 2.

**Table 2:** Frequency of on-treatment complications of decompensated cirrhotic HCV patients treated with Sofosbuvir-based regimens (n = 150).

Complication	(%)
Anemia	26 (17%)
Renal dysfunction	21 (14%)
Increased aminotransferases	1 (0.7%)
Cardiac arrhythmia	1 (0.7%)
Worsening of the previous ascites	20 (19%)
Hepatic encephalopathy	8 (5%)
New-onset ascites	6 (4%)
Variceal upper gastrointestinal hemorrage	5 (3%)
Hepatocellular carcinoma	2 (1%)
Infections	35 (23%)
Pneumonia	10
Skin and soft tissue	8
Urinary tract	7
Spontaneous bacterial peritonitis	1
Death	9

Regarding tolerability, the most frequent adverse event observed was anemia, which occurred in 26 (17%) patients. Among patients who developed anemia, 73% (19/26) used ribavirin. Anemia was resolved with a dose reduction of ribavirin in 48% of cases and withdrawal in the remaining 52%. Discontinuing DAA was not required in any case of anemia, nor did there a need for hematopoietic stimulating factors or blood transfusion.

Twenty-one (14%) patients experienced renal dysfunction; among these, 18 (86%) had a previous diagnosis of essential arterial hypertension or diabetes. Most renal dysfunction cases were mild (AKI I in 17 patients). One patient discontinued treatment due to severe acute renal dysfunction and was referred to hemodialysis. One patient presented increased aminotransferase (5× upper limit of normal) of unknown cause with spontaneous resolution. Discontinuation of treatment was not necessary for these patients. One patient was hospitalized for tachyarrhythmia, documented as atrial fibrillation, and successfully treated. No relationship was established with DAA treatment, and the medications were maintained.

Infectious were the most common complications during treatment. Thirty-five (23%) patients presented at least 1 infectious event. The most frequent source of infection was the respiratory tract (29%), followed by skin and soft tissue (23%) and urinary tract (20%). Only 1 patient developed a single episode of spontaneous bacterial peritonitis (SBP). *Predictive factors of cirrhosis decompensation*: Although 75% of cases were minor and controlled with antibiotics on an outpatient-based schedule, infections were the leading cause of death during treatment (78%).

Thirty-three patients (22%) had at least 1 episode of decompensation of cirrhosis throughout treatment. The most common outcome of cirrhosis decompensation was the worsening of previous ascites in 19%, defined as increased diuretic doses or the need for paracentesis: only 4% of patients presented with a new episode of ascites during treatment. Hepatocellular carcinoma was diagnosed in 2 patients after 84 and 141 days of treatment.

Nine patients died due to the following complications: sepsis (7), pulmonary thromboembolism (1), and hemorrhagic stroke (1).

### 3.3. Predictive factors of cirrhosis decompensation and infection during treatment

**Figure 1.**
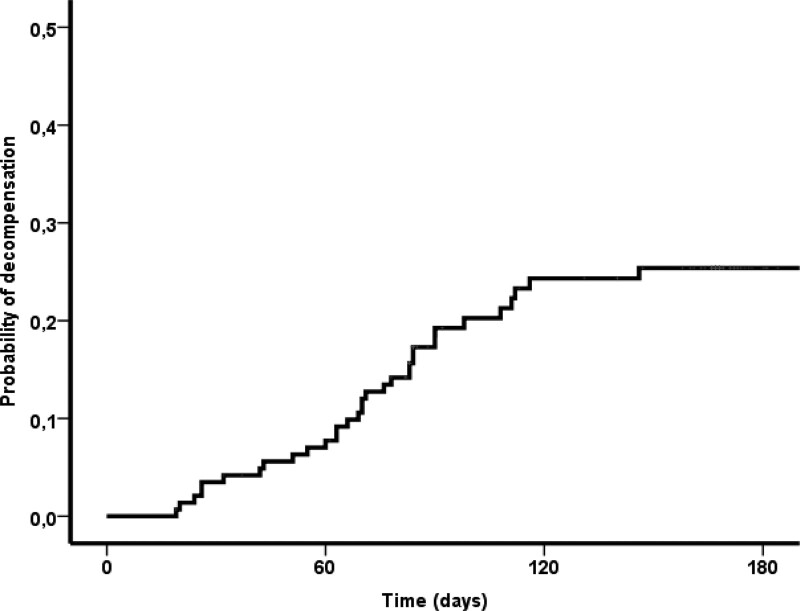
Probability of decompensation during treatment.

**Figure 2.**
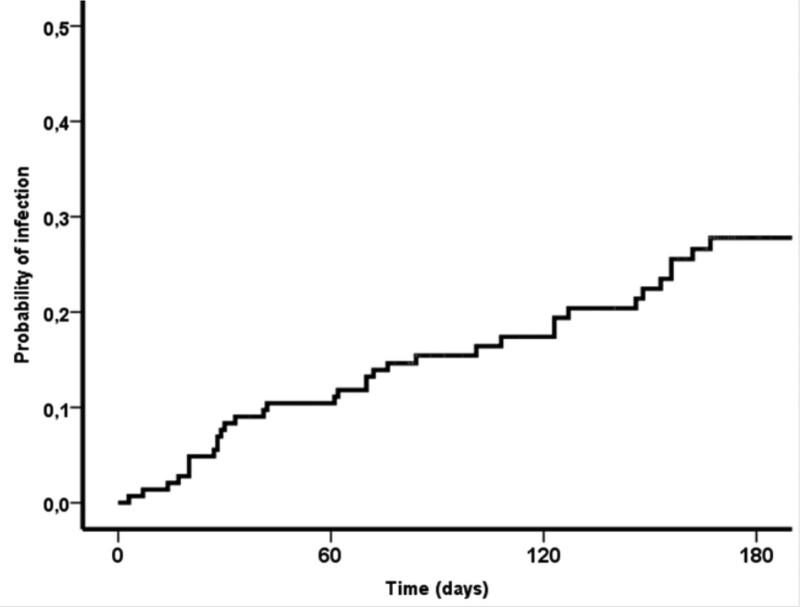
Probability of infection during treatment.

The probability of decompensation in 28, 90, and 180 days was 4%, 19%, and 25% (Fig. 1). At univariate analysis, baseline platelet levels (OR 0.992, 95% CI 0.983–1.00, *P* = .08), CHILD ≥ B8 (OR 0.53; 95% CI 0.26–1.07; *P* = .08) showed a trend toward cirrhosis decompensation during treatment. Infection during treatment (OR 2.3; 95% CI 1.11–4.72; *P* = .03) was significantly associated with decompensation in the same period (Table 3). In a multivariate analysis, the only independent factor that predicted cirrhosis decompensation during treatment was infection (OR 2.24; 95% CI 1.09–4.61; *P* = .03). The probability of infection in 28, 90, and 180 days was respectively 7%, 15.4%, and 27.7% (Fig. 2). At univariate analysis, baseline bilirubin levels (OR 1.56; 95% CI 1.12–2.18; *P* < .01), MELD (OR 1.16, 95% CI 1.07–1.25; *P* < .001), and CHILD ≥ B8 (OR 0.33; 95% CI 0.16–0.7; *P* < .01) were significantly associated with infection during treatment (Table 4). A multivariate analysis, both baseline MELD (OR 1.13; 95% CI 1.03–1.24, *P* = .01)0.45 (0.21–0.97) 0.043 and CHILD ≥ B8 (OR 2.22; 95% CI 1.03–4.76; *P* = .43) were independently associated with infection during treatment.

**Table 3:** Univariate and Multivariate Predictors of decompensation during treatment.

Covariate	Univariate		Multivariate	
	OR (95% CI)	*P* value	OR (95% CI)	*P* value
Age	1.029 (0.98–1.07)	0.18	–	–
Esophageal varices	1.707 (0.52–5.6)	0.38	–	–
Propranolol	0.75 (0.37–1.53)	0.43	–	–
Diabetes	0.91 (0.45–1.87)	0.81	–	–
Albumin (g/dl)	1.2 (0.63–2.29)	0.57	–	–
Bilirubin (mg/dl)	1.2 (0.83–1.76)	0.32	–	–
Platelet count (×10^9^/mm³)	0.99 (0.98–1.00)	0.08	–	–
Creatinine (mg/dl)	0.92 (0.54–1.59)	0.77	–	–
Sodium (mEq/L)	1.0 (0.91–1.11)	0.92	–	–
MELD	1.03 (0.93–1.14)	0.54	–	–
Ascitis	0.86 (0.43–1.71)	0.66	–	–
Hepatic encephalopathy	0.65 (0.31–1.37)	0.26	–	–
Baseline Child-Pugh [scolor_start FADADD]≥[/scolor] B8	0.53 (0.26–1.07)	0.08	–	–
Infections during treatment	2.3 (1.11–4.72)	0.03	2.24 (1.09–4.61)	0.03

**Table 4:** Univariate and multivariate predictors of infection during treatment.

Covariate	Univariate		Multivariate	
	OR (95 CI)	*P*-value	OR (95 CI)	*P* value
Age	0.97 (0.94–1.01)	0.12	–	–
Esophageal varices	1.87 (0.85–4.2)	0.12	–	–
Propranolol	0.73 (0.36–1.45)	0.37	–	–
Diabetes	1.12 (0.56–2.23)	0.75	–	–
Albumin (g/dl)	0.81 (0.42–1.6)	0.53	–	–
Bilirubin (mg/dl)	1.56 (1.12–2.18)	<0.01	–	–
Platelet count (x10^9^/mm³)	1.00 (0.99–1.01)	0.55	–	–
Creatinine (mg/dl)	1.14 (0.81–1.59)	0.45	–	–
Sodium (mEq/L)	0.99 (0.91–1.1)	0.96	–	–
MELD	1.16 (1.07–1.25)	<0.01	1.13 (1.03–1.24)	0.01
Ascitis	1.13 (0.56–2.21)	0.72	–	–
Hepatic encephalophaty	1.91 (0.94–3.85)	0.07	–	–
Baseline Child-Pugh [scolor_start FADADD]≥[/scolor] B8	0.33 (0.16–0.7)	<0.01	2.22 (1.03–4.76)	0.04

## 4. Discussion

This prospective cohort of 150 patients with decompensated HCV-related cirrhosis study showed that treatment with a sofosbuvir-based regimen is effective and safe. Nevertheless, liver-related decompensation and infections were frequent, the latter being the leading cause of death in this real-life study. We observed that infection is an independent predictor of liver-related decompensations, and Child-Pugh and MELD scores at the baseline were related to infection during treatment.

Several studies reported the efficacy of DAAs in cirrhotic patients.^[25,26]^ The improvement in outcomes is unquestionable essentially in compensated cirrhotics.^[25]^ Few real-world data concerning the safety and efficacy of DAA regimens for HCV treatment in Brazil or Latin America were reported.^[27–30]^ None of these studies were dedicated to evaluating the population of decompensated cirrhotics. Lobato et al performed a study involving 20 centers across Brazil to study the effectiveness and safety of HCV treatment in all patients with advanced fibrosis and cirrhosis.^[30]^ Among 3939 included patients, 236 (6%) were decompensated cirrhotics. However, neither the characteristics of this specific group nor their related outcome was reported. Our study is the largest cohort composed exclusively of decompensated cirrhotics in Latin America and the second-largest when considering the published real-life studies, lagging behind the British study by Foster et al,^[9]^ which included 406 patients.

In our study, we achieved 91% of SVR by intention-to-treat analysis. Although this finding was a striking result, it is less than that observed in the study of Lawitz et al, a phase 2 trial that did not include CHILD C patients and reported a 100% rate of SVR12 in patients with decompensated liver disease.^[31]^ This study found that most patients had a MELD below 10 (55%). Thus, compared to our population, there was a higher prevalence of patients with advanced disease in the present study since 8% were CHILD C and only 24% had a MELD under 10. The second study from Gentile et al demonstrated a higher SVR than the present study.^[12]^ They found an SVR of 95.5% in a smaller population of 89 patients, including only Child B patients and only 1 with genotype 3. A multicenter Egyptian study that included a significant number of genotype-4 cirrhotic patients observed an SVR of 96% among Child-Pugh B patients, corroborating the high response rate characterized by Child-Pugh.^[32]^ Genotype-4 is rare in Brazil, and we did not have any genotype-4 patients in our study.^[33]^ This way, we cannot compare this SVR rate with the 1 from the present study.

In contrast, the Australian study,^[11]^ which used the sofosbuvir plus daclatasvir regimen like ours predominantly, found an RVS12 of 70% by intention-to-treat analysis. This result is much lower than ours and can be explained by the Australian study that included even more severe patients with MELD higher than 15, while only 28% had a MELD score equal to or greater than our population. Moreover, genotype-3 was the most common in this Australian population, whereas, in our study, most patients (84%) were genotype 1. Recently, it was demonstrated that an SVR of 85% was achieved in the French HEPATHER cohort.^[19]^ It comprised 559 patients, most Child-Pugh A, with past liver-disease decompensation and 37% Child-Pug score B. Overall, 55 patients (6%) presented with *The associated infection episodes resulted in 66%* or MELD score > 20. The SVR could not be analyzed in this group due to the small number of patients. In Brazil, only the study from Lobato et al has shown an SVR12 of 85% in the group of decompensated cirrhosis.^[30]^

The most common adverse events were mild fatigue, insomnia, and headache. Because they were already widely explored and corroborated by various additional studies, we did not discuss them. We instead evaluated the occurrence of serious adverse events that could endanger the maintenance of treatment and SVR rate. Among the patients studied, increased aminotransferase levels and arrhythmia were irrelevant since they did not impact the maintenance of treatment or SVR.

Anemia occurred in 73% of patients using ribavirin and improved with dose reduction or withdrawal. This fact was also observed by Modi et al,^[14]^ who showed that 43% of the patients using ribavirin developed anemia. In that study, 1 patient needed a blood transfusion, and another needed a dose reduction of ribavirin. Foster et al^[9]^ used a narrower definition of significant anemia (Hb < 80g/ L) and still described the event in 5.4% of patients who received ribavirin. However, no dose reduction of ribavirin or treatment discontinuation was reported.

Our study noticed a significant frequency of acute kidney injury in 21 patients (14%). However, in 17 patients, it corresponded to mild injury characterized as AKI I. Only 1 patient had a more severe condition requiring treatment discontinuation, dialytic support and later complicated with pulmonary sepsis and death. These outcomes were not a frequent finding in other studies. Foster et al10 demonstrated that acute kidney injury was an uncommon finding, present in only 2.8% of patients, but once again used a strict criterion defining the event as the presence of creatinine greater than or equal to 1.5 mg in the twelfth week of treatment.

We observed a significant frequency of infections during treatment. This finding has not been much explored in other studies, perhaps because the literature shows that infections probably correspond to the natural course of the evolution of decompensated cirrhosis.^[34,35]^ The risk of bacterial infection in cirrhosis is caused by multiple factors, including liver dysfunction, portosystemic shunting, gut dysbiosis, increased bacterial translocation, cirrhosis-associated immune dysfunction, and genetic factors.^[36]^ This immune defect facilitates bacterial translocation, induced by increased intestinal permeability and gut bacterial overgrowth observed in cirrhosis. Genetic immune defects can contribute to the high risk of bacterial infections in cirrhosis, particularly SBP.^[36,37]^ Indeed, the development of bacterial infections may accelerate the course of the disease at any stage, especially in decompensated cirrhosis.^[38]^

Saxena et al^[17]^ 18 reported infections in 20% (11/55) of patients, with the primary source being soft tissue infection (42%). The associated infection episodes with 66% of early treatment discontinuation and 50% of hospitalizations, but no deaths were described due to infection. Among 2 patients who died in their study, 1 was due to cirrhosis decompensation and the other to a lymphoma. We had a similar frequency (23%) of infection. However, in our study, infection was the leading cause of death.

Except for the present study, Saxena was also the only one to evaluate predictors of hepatic decompensation and found that in this population, Child B/C was the only significantly associated predictor.^[17]^ His analysis did not include infections but Child B/C, bilirubin, INR, albumin, platelet, hepatic encephalopathy, and ascites. In addition to these variables, our study also analyzed the occurrence of infections during treatment and showed that infections were the only independent predictor of decompensation. However, when studying the predictors of infection, we also found CHILD ≥ B8 and MELD as independent predictive factors.

As with most real-life studies, our work has some limitations. Our cohort has a relatively small number of patients, and we used a convenience sample, following strict inclusion and exclusion criteria. The treating physician decided on treatment duration and adding or not ribavirin and may not have strictly followed the treatment recommendations. Despite the commitment of the study participants, there could be an under-reporting of adverse events or clinical decompensations. Treatment adherence may not be adequately evaluated. Also, patients infected with genotypes 2 and 4 are underrepresented in the present study, making it challenging to generalize current findings to these patients.

In summary, this was a significant, real-life study of all-oral HCV Therapy in a large cohort of patients with decompensated cirrhotics. The overall virological response was high, and the adverse events were primarily mild, although the frequency of liver-associated decompensation and infections was high. The longer-term benefits of DAA therapy in patients with decompensated cirrhosis remain to be ascertained.

## Author contributions

Lívia Victor: study concept and design; study supervision; analysis and interpretation of data; drafting and critical revision of the manuscript. Juliana Piedade: data collection; critical revision and approval of the final version of the manuscript. Flávia Fernandes: study supervision; analysis and interpretation of data; critical revision and approval of the final version of the manuscript; Renata Perez, Cristiane Villela-Nogueira, Gustavo Pereira: study concept and design; study supervision; analysis and interpretation of data, critical revision and approval of the final version of the manuscript.
